# Clinical and immunological characteristics of prolonged SARS-CoV-2 Omicron infection in hematologic disease

**DOI:** 10.1038/s41408-023-00897-5

**Published:** 2023-09-05

**Authors:** Daisuke Ikeda, Ami Fukumoto, Yuka Uesugi, Rikako Tabata, Daisuke Miura, Kentaro Narita, Masami Takeuchi, Tomohisa Watari, Yoshihito Otsuka, Kosei Matsue

**Affiliations:** 1 Division of Hematology/Oncology, Department of Medicine, Kameda Medical Center, Kamogawa-shi, Japan; 2 Central laboratory, Kameda Medical Center, Kamogawa-shi, Japan

**Keywords:** Preventive medicine, Haematological cancer

**Dear Editor**,

Prolonged viral shedding (PVS) results from a failure of viral eradication. Before the emergence of SARS-CoV-2 Omicron variant, PVS was reported mainly in patients with hematologic disease (HD), posing significant concerns regarding patient outcomes and public health [1, 2]. Lee et al. showed that 13.9% (51/368) of patients infected with SARS-CoV-2 developed PVS, and among the evaluable cases with persistent infection, 26.3% (5/19) died [2]. Moreover, they showed that the combined depletion of B and CD4 + T-cells played a dominant role in viral persistence. Nevertheless, although several studies have reported the incidence of PVS [3, 4], knowledge integrating the clinical and immunological characteristics of PVS in the Omicron era is limited. This study aimed to identify the risk factors for Omicron PVS and to profile the associated immune deficits in a cohort with HD.

We conducted a retrospective analysis of patients with HD who developed laboratory-confirmed breakthrough COVID-19 infection from January 2022 to 2023, when Omicron was the predominant variant in Japan. Using reverse transcription-PCR, PVS was defined as cycle threshold value (Ct) < 30 for 21 days or more after the onset of COVID-19 according to previous studies [1]. In subgroups with available samples, Omicron-specific neutralizing effects were evaluated using competitive enzyme-linked immunosorbent assay, and T-cell phenotype was analyzed to classify activated (CD38^high^ /HLA-DR^high^) and exhausted T-cells (PD-1^high^). Detailed methods regarding study design are described in Supplementary method.

Of the 1197 consecutive patients with HD (Supplementary Table 1), 160 (13.3%) developed an Omicron breakthrough infection throughout one year by January 2023 (Table 1). Overall, 139 patients (86.9%) had mild or asymptomatic symptoms, 95 (68.3%) of which resolved spontaneously. Ritonavir-boosted nirmatrelvir was the most used initial anti-viral agent, administered to 21 of the 65 (32.3%) patients. Twenty-one patients (13.1%) developed severe illness. The hospitalization rate reached 21.3%; however, most cases resolved after initial anti-viral therapies, and death attributable to COVID-19 occurred in only four patients (2.5%).

**Table 1:** Clinical characteristics of patients with COVID-19 according to disease severity.

	Total patients with COVID-19 (*n* = 160 [100%])	Asymptomatic/mild (*n* = 139 [86.9%])	Severe/critical (*n* = 21 [13.1%])	*P*-value
Age, years, median (IQR)	68 (57–78)	66 (55–77)	78 (71–82)	0.001
≧70, *n* (%)	71 (44.4)	55 (39.6)	16 (76.2)	<0.001
Female, *n* (%)	62 (38.8)	58 (41.7)	4 (19.0)	0.08
ECOGPS ≧ 2, *n* (%)	22 (13.8)	14 (10.1)	8 (38.1)	0.002
Modified CCI except malignancy ≧ 2, *n* (%)	24 (15.0)	13 (9.4)	11 (52.4)	0.004
Relapsed or refractory condition, *n* (%)	28 (17.5)	20 (14.4)	8 (38.1)	0.018
Background hematological disease, *n* (%)				
Lymphoid neoplasms	108 (67.5)	90 (64.7)	18 (85.7)	0.078
B-cell lymphoma	64 (40.0)	50 (36.0)	14 (66.7)	0.015
Plasma cell dyscrasia	28 (17.5)	25 (18.0)	3 (14.3)	1
Others	16 (10.0)	15 (10.8)	1 (4.8)	0.69
Myeloid neoplasms	35 (21.9)	34 (24.4)	1 (4.8)	0.047
Benign hematologic disorders	17 (10.6)	15 (10.8)	2 (9.5)	1
Treatment of underlying disease, *n* (%)				
Ongoing treatment	42 (26.2)	20 (14.4)	8 (38.1)	0.018
Anti-CD20 antibody within 2 years	41 (25.6)	28 (20.1)	13 (61.9)	<0.001
Bendamustine within 2 years	11 (6.9)	6 (4.3)	5 (23.8)	0.005
HSCT within 2 years	2 (1.2)	2 (100)	0 (0)	—
Time distribution of infection, *n* (%)				0.067
January–March 2022	26 (16.2)	20 (14.4)	6 (28.6)	—
April–June 2022	6 (3.8)	4 (2.9)	2 (9.5)	—
July 2022–January 2023	128 (80.0)	115 (82.7)	13 (61.9)	—
Vaccination doses before infection, *n* (%)				
Two	43 (26.9)	35 (25.2)	8 (38.1)	0.289
≧Three	117 (73.1)	104 (74.8)	13 (61.9)	—
Bivalent vaccination against the Omicron variant	21 (13.1)	20 (14.4)	1 (4.8)	0.31
Pre-exposure prophylaxis with tixagevimab–cilgavimab	1 (0.6)	0 (0.7)	0 (0)	—
Anti-S level after infection, U/mL, median (IQR)^a^	6705 (87.3–50118)	14650 (758–52614)	18.7 (0.4–866)	< 0.001
Seronegative after infection, *n* (%)^a^	13 (9.5)	6 (5.1)	7 (35.0)	<0.001
Initial COVID-19 treatment, *n* (%)				<0.001
RDV	19 (11.9)	13 (9.4)	6 (28.6)	—
RDV + DEX	4 (2.5)	0 (0)	4 (19.0)	—
RDV + DEX+Tocilizumab	1 (0.6)	0 (0)	1 (4.8)	—
Ritonavir-boosted nirmatrelvir	21 (13.1)	20 (14.4)	1 (4.8)	—
Molnupiravir	10 (6.2)	7 (5.0)	3 (14.3)	—
Sotrovimab	6 (3.8)	4 (2.9)	2 (9.5)	—
Steroid pulse	1 (0.6)	0 (0)	1 (4.8)	—
Untreated	98 (61.2)	95 (68.3)	3 (14.3)	—
COVID-19 severity, *n* (%)				< 0.001
Asymptomatic	19 (11.9)	19 (13.7)	0 (0)	—
Mild	120 (75.0)	120 (86.3)	0 (0)	—
Severe	16 (10.0)	0 (0)	16 (76.2)	—
Critical	5 (3.1)	0 (0)	5 (33.8)	—
Outcome, *n* (%)				
Hospitalization	34 (21.3)	13 (9.4)	21 (100)	< 0.001
Death attributable to COVID-19	4 (2.5)	0 (0)	4 (19.0)	<0.001
Death due to other cause	6 (3.7)	1 (0.7)	5 (23.8)	< 0.001
Death due to progression of underlying HD	5 (3.1)	1 (0.7)	4 (19.0)	0.001

*IQR* interquartile range, *ECOGPS* European Cooperative Oncology Group Performance Status, *CCI* Charlson Comorbidity Index, *HSCT* hematopoietic stem cell transplantation, *anti-S* antibody against spike receptor binding domain, *COVID-19* coronavirus disease 2019, *RDV* remdesivir, *DEX* dexamethasone, *HD* hematologic disease.

^a^Anti-S after infection was evaluable in 137 patients.

Longitudinal Ct assessment was performed in 63 (39.4%) patients, of these, 17 were excluded due to inconclusive data confirming PVS; therefore, 46 (28.7%) patients were analyzed (Supplementary Fig. 1). This subgroup includes a significantly higher proportion of hospitalized patients (45.6% vs. 25.6%, *P* = 0.011) and those who received anti-CD20 antibodies within two years (39.1% vs. 21.3%, *P* = 0.02) compared to the original cohort (Supplementary Table 2). The Ct changes in these patients are shown in Fig. 1A. The median interval from COVID-19 onset to the first follow-up PCR testing was 17 days (range: 6–23). PVS was observed in 17 (36.9 %) patients. Six (35.3%) patients did not achieve viral clearance even at 42 days after symptom onset, with the longest duration of 85 days. Five of the six patients had previously received bendamustine plus anti-CD20 antibodies, and the remaining patient on Bruton’s tyrosine kinase inhibitor for chronic lymphocytic leukemia developed PVS despite a second booster dose of a bivalent vaccine against Omicron. Next, lymphocytes, B-cells, and T-cells counts after infection were compared. CD4 + and CD19+ cells were significantly depleted in patients with PVS compared to those without (CD4 + , median 174/μL IQR [75–290] vs. 409/μL [216–555], *P* = 0.001; CD19 + , 0/μL [0–9] vs. 37/μL [0–157], *P* = 0.004) but absolute lymphocyte counts, and CD8+ cells were not. Univariate analysis showed that a low number of CD4+ cells and CD19+ cell, recent anti-CD20 antibodies and bendamustine, seronegative, and severe disease were associated with an increased risk of PVS (Supplementary Table 3).

**Fig. 1:**
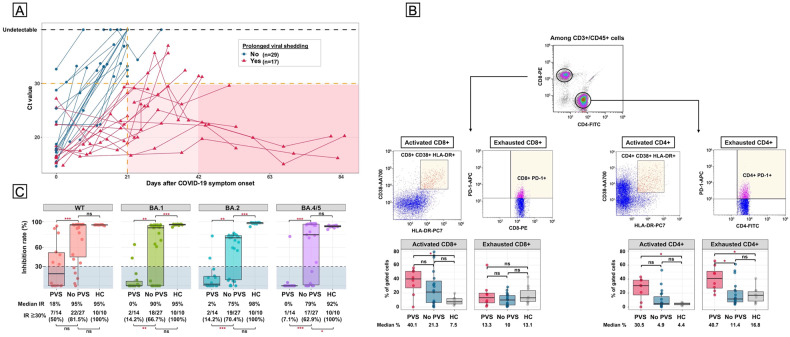
**Ct values of SARS-CoV-2 PCR, T-cell phenotype, and neutralizing activity to three Omicron variants after infection.** **A** Longitudinal changes in cycle threshold (Ct) values after the onset of COVID-19 symptoms. Red lines with triangles, patients with prolonged viral shedding; blue lines with circles, patients without prolonged viral shedding. Red-shaded area, duration of persistent PCR positivity; deep red, positive PCR results beyond 42 days. **B** Phenotypic T-cell profiling after infection. The upper part shows the gating strategy, in which CD4+ and CD8+ cells are selected from CD3 + /CD45+ cells, followed by the identification of activated and exhausted T-cells based on the expression of CD38^high^/HLA-DR ^high^ and PD-1 ^high^ markers within each subset. The lower part of the figure shows a box plot comparing the percentages of activated and exhausted T-cells within CD4+ and CD8 + T-cells in patients with prolonged viral shedding versus those without and healthy controls. Abbreviations: PVS, prolonged viral shedding; HC, healthy control; ns, not significant. *P < 0.05. **C** Neutralizing effects against wild-type and three Omicron subvariants after infection. The boxplots depict the inhibition rates (IRs) against recombinant spike proteins for the wild-type, BA.1, BA.2, and BA.5 subvariants after infection among patients with or without prolonged viral shedding and healthy controls. Blue shaded area, IR < 30%. Abbreviations: WT, wild-type; PVS, prolonged viral shedding; HC, healthy control; IR, inhibition rate; ns, not significant. *P < 0.05, **P <0.01, ***P < 0.001.

To further characterize the T-cell immunity associated with PVS, T-cell phenotype was analyzed on 17 patients (seven with PVS and ten without PVS) and ten healthy controls (HCs) with contracted infections (Supplementary Table 4). The frequency of exhausted T-cells with high PD-1 expression within CD4 + T cells was significantly higher in PVS than in non-PVS and HC (median 40.7% vs. 11.4% vs. 10.8, *P* = 0.019), but similar in CD8 + T-cells (13.3% vs. 10% vs. 13.1%, *P* = 0.96) (Fig. 1B). Regarding T-cell activation, there was a non-significant trend of increased proportions of CD38^high^ /HLA-DR^high^ T-cells within CD4+ cells (median 30.5% vs. 4.9%, *P* = 0.059), but not within CD8 + T-cells (40.1% vs. 21.3%, *P* = 0.328) in PVS compared to non-PVSs.

Forty-one patients (14 with PVS and 27 non-PVS) and 10 HCs were analyzed for assessment of neutralizing activities (Fig. 1C). The rate of positive neutralizing effects against the wild-type did not differ significantly (PVS 50% vs. non-PVS 81.5%, *P* = 0.067). However, patients with PVS were significantly less likely to achieve neutralization against all subvariants compared to those without PVS (BA.1: 14.2% vs. 66.7%, *P* = 0.002; BA.2: 14.2% vs. 70.4%, *P* < 0.001; BA.5: 7.1% vs. 62.9%, *P* < 0.001).

Finally, the clinical course and viral status of the 17 patients previously treated with bendamustine plus anti-CD20 antibodies are shown in Supplementary Fig. 2 and Supplementary Table 5. Among the four patients (patients 1–4) who died of COVID-19, all had a history of combination therapy. Notably, late-onset interstitial pneumonia (IP) was observed in three patients (17.6%), two of whom were negative by PCR at the time of IP development. In patient 2, aggressive immunosuppressive therapy combined with anti-viral retreatment led to transient viral reappearance and failed to prevent the rapid progression of pulmonary fibrosis (Supplementary Fig. 3). Additionally, seven patients were diagnosed with PVS, including two critical cases (patients 1 and 3). Among these, three patients (patients 7, 9, and 10) experienced recrudescent fever and required retreatment with anti-viral therapies. In contrast, the remaining four patients (patients 5, 8, 10, and 14) did not experience recurrent symptoms related to COVID-19 after initial treatment.

PVS was primarily reported during the pre-Omicron era, with rates ranging from 13.9% to 25.4% [1, 2] but few studies have focused specifically on Omicron (17% to 27.9%) [3, 4]. In our study, the higher frequency (36.9%) of PVS may be attributed to variations in the definition of PVS, and the method used to confirmation, and the larger number of evaluable patients with recent B-cell depletion (45.6%). B- and CD4 + T-cell depletion, in addition to immunochemotherapy, were significant determinants of the increased risk of PVS. This finding is consistent with the largest series reported for ancestral variants [2]. In hematologic malignancies and cancers, early reports showed that a decreased function of T-cells occurred due to the disease itself or its treatment even before infection [5] and worsened with disease severity after infection [6, 7]. The association between CD4 + T-cell exhaustion and PVS observed in this study may reflect the severity of patients who develop PVS. Furthermore, the neutralizing effect was attenuated in patients with PVS, particularly against the Omicron variants. These findings align with previous studies in macaques, highlighting the complementary relationship between neutralizing antibodies, cellular immunity, and protection [8]. Altogether, despite the distinct biological properties of Omicron, such as its tissue tropism into the upper airway and potentially lower virulence [9], the host immune system associated with viral clearance may be preserved between Omicron and earlier and possibly future variants.

The COVID-19 mortality rate reported in this study was comparable to those reported in the most recent EPICOVIDEHA report (3.9%) [10] and other studies (3.4%) [3]. The wide availability of newer anti-viral drugs, such as nirmatrelvir/ritonavir, may improve outcomes [11]. Whether ongoing infection, induced inflammation, or both are the leading drivers of late-onset IP remains unclear [12]. A notable case (patient 2) may provide a speculative explanation, suggesting that persistent infection in the lung, undetectable in the nasopharynx, may contribute to IP pathogenesis. Recently, cases have demonstrated successful eradication of the virus and prevented organized pneumonia in patients with prolonged COVID-19 infection through strong anti-viral therapy, including the use of remidesivir and nirmatrelvir/ritonavir with or without sotrovimab [13]. We hypothesized that the residual virus in the lower respiratory tract and the production of inflammatory cytokines in response may contribute to the development of organizing pneumonia with fibrosis. Although early intervention with corticosteroids is effective in reversing pulmonary functional deficits [14], the use of high-dose corticosteroids is likely to compromise the potency of anti-viral therapy. The optimal use of corticosteroids and other immunocompromising agents in patients with severe immunosuppression requires further investigation.

This study has several limitations that should be addressed in future studies. First, the retrospective design and small sample size precluded multivariate analysis from weighing the relative contributions of B- and T-cell depletion to viral persistence. The prevalence of PVS might be overestimated due to potential selection bias. Neutralizing activity was assessed using an ELISA-based surrogate virus neutralization test [15]. Finally, the absence of genomic sequencing prevented the detection of escape mutations and the definitive differentiation of possible reinfection from persistent infection.

In conclusion, this study suggests that Omicron breakthrough infection may be associated with a relatively high frequency of PVS. We also identified the risk factors for PVS, with particular emphasis on the combined use of CD20 monoclonal antibodies and bendamustine. B-cell depletion, CD4 + T-cell exhaustion, and impaired neutralization to the Omicron variant may be associated with the developing PVS following infection. Delayed IP is a potentially fatal complication. Despite the use of high-dose corticosteroids and effective anti-virals, the optimal treatment for PVS remains unclear and should be individualized until a more effective strategy is established.

### Supplementary information

Supplemental materials

## Data Availability

The datasets generated and analyzed during the current study are available from Daisuke Ikeda or Kosei Matsue on reasonable request.
