# Supplementary material for: Clinical and immunological characteristics of prolonged SARS-CoV-2 Omicron infection in hematologic disease
Source: Blood Cancer J. 2023 Sep 5;13(1):133. doi: 10.1038/s41408-023-00897-5 (PMC10477167; doi:10.1038/s41408-023-00897-5)

Supplementary materials

Supplementary methods

Patient cohort and study design

We previously performed a prospective observational cohort study to investigate the incidence and clinical outcomes of Omicron breakthrough infection and serological response after mRNA-based vaccination in 922 patients with HD from January to September 2022.[1] For the retrospective analysis of laboratory-confirmed COVID-19 cases, this study was conducted with expanded eligibility to include outpatients without serological data and an extended study period of up to January 2023. The severity of COVID-19 was graded according to the EPICOVIDEHA survey.[2] The present study was conducted in accordance with the Declaration of Helsinki and was approved by our institutional review board (approval number:22-079).

Diagnosis and epidemiological trends of COVID-19

COVID-19 was confirmed by polymerase chain reaction (PCR) or quantitative antigen testing. In Japan, national epidemiological data have shown the predominance of Omicron since January 2022, with the emergence of several subvariants, including BA.1 (from January to March), BA.2 (from April to June), and BA.5 (from July).[3] Accordingly, the causative variant during the study period was assumed to be Omicron. In particular, BA.5 lineages rapidly replaced the other strains from July 2022 and continued to be mainstream. As of January 31, 2023, BA.5 lineages, including BQ.1 (sublineages of BA.5.3), remained dominant and accounted for approximately 80% of COVID-19 cases in national genome surveillance, whereas the proportion of BA.2 lineages, including BA.2.75, increased to approximately 15%.[4] Meanwhile, XBB lineages, reported mainly in the United States, were first detected in December 2022 but did not spread during the study period. COVID-19 cases diagnosed elsewhere were tracked via questionnaires or telephone interviews conducted on March 31, 2023.

Reverse transcription-PCR (RT-PCR) testing and definition of PVS

Reverse transcription-PCR of the ORF, N, and S genes was performed on nasopharyngeal specimens using a TaqPath test kit on a QuantStudio 5 Dx instrument (Thermo Fisher Scientific, MA, USA). The cycle threshold value (Ct) is the number of cycles required to amplify viral RNA to a detectable level and is used to estimate the viral load.[5] Early reports from the pre-Omicron era showed that all virus samples with Ct >30 were unculturable;[6] therefore, Ct >30 for the ORF was considered a negative PCR result in this study. As there are no established criteria for the duration of positive PCR results, we defined PVS as persistent PCR positivity for 21 days or more after the onset of COVID-19, according to previous studies.[7] Patients with

indeterminate PCR results; that is, Ct <30 before 21 days but not followed up, or who achieved negativity after 21 days, were excluded from the analysis of viral kinetics.

S-gene target failure (SGTF) is a genetic signature that serves as a reliable proxy for specifically distinguishing the BA.1 and BA.5 lineages from BA.2 lineages.[8] As mentioned above, owing to the predominant circulation of BA.5 since July 2022, patients with COVID-19 with SGTF during this period were predicted to have BA.5 lineage infections.

Although viral genomic analysis is considered the gold standard for differentiating PVS from reinfection, it is not widely available for clinical practice. Alternatively, time-based definitions for reinfection are often applied. Based on the definition proposed by the Centers for Disease Control and Prevention, investigation for reinfection is considered when the detection of SARS-CoV-2 RNA occurs ≧45 days after the first detection.[9] Therefore, the interval between the first PCR test and the follow-up test was described to assess the degree to which reinfection could be misinterpreted as PVS.

Phenotypic T-cell profiling using multiparametric flow cytometry

Among patients and healthcare workers infected during the same observation period, individuals with available stored plasma samples were included in the T-cell profile analysis. Peripheral mononuclear blood cells after infection were collected and preserved at −80°C until analysis. The antibodies used for staining were CD45-KrO, CD3-AA750, CD4-FITC, CD8-PE, CD38-AA700, human leukocyte antigen (HLA)-DR-PC7, and programmed death-1 (PD-1)-APC. Of the cells gated with CD45+/CD3+, those with CD38^{high} /HLA-DR^{high} were profiled as “activated T-cells” and those with PD-1^{high} as “exhausted T-cells,” based on preceding studies.[10] Data were acquired on a Navios flow cytometer using Kaluza software (Beckman Coulter, CA, USA).

Evaluation of the neutralizing effects against Omicron variants

Omicron-specific neutralizing effects were evaluated by competitive enzyme-linked immunosorbent assay (ELISA) (GTX537233, GeneTex, CA, USA) using a Bio-Rad ELISA microplate reader (Bio-Rad Laboratories, CA, USA). Serum obtained from infected individuals was added to a 96-well plate coated with competitive recombinant angiotensin-converting enzyme-2 (ACE-2) proteins, followed by the addition of histidine (His)-tagged recombinant trimeric spike proteins from the wild-type strain and three Omicron subvariants (BA.1, BA.2, and BA.5). After incubation and washing, a horseradish peroxidase-conjugated anti-His-tag antibody and tetramethylbenzidine substrate were added to elicit chromogenic signals. The neutralizing effect was inversely correlated with signal intensity and was represented as the inhibition rate

(IR). The IR was calculated using the following formula: (1 - average absorbance value of tested sample/average absorbance value of negative control) × 100. An IR of ≧30% was considered evidence of positive neutralizing effects according to the manufacturer's instructions. Before examination, neutralization pre- and post- administration of tixagevimab–cilgavimab, which retains significant in vitro activity against BA.1, BA,2, and BA.5,[11] was assessed in an uninfected patient who remained seronegative even after four vaccine doses. Before and after receiving the monoclonal antibody, the IR increased from 0% to >90% across the wild-type and all subvariants (data not shown), supporting the applicability of this assay. Anti-SARS-CoV-2 spike receptor binding domain antibodies (anti-S) were also measured (Roche Diagnostics, Burgess Hill, UK) with seronegativity defined as <0.8 U/mL and a detectable limit of 0.4 U/mL, as described previously.[12]

Statistical analysis

Continuous variables were analyzed using the Mann–Whitney U and Kruskal–Wallis tests, as appropriate, whereas categorical variables were compared using Fisher's exact test. Clinical and laboratory factors associated with an increased risk of PVS were evaluated by univariate logistic regression analysis. All statistical analyses were performed using R version 4.1.1 (R Foundation, Vienna, Austria). Statistical significance was defined as a two-sided P-value of < 0.05.

Referrences

1. Narita K, Ikeda D, Seki M, Fukumoto A, Tabata R, Uesugi Y, et al. Prevalence and Clinical Outcome of Omicron Breakthrough Infection in Patients With Hematologic Disease: A Prospective Observational Cohort Study. Hemasphere. 2023;7(6):e905.

2. Pagano L, Salmanton-Garcia J, Marchesi F, Busca A, Corradini P, Hoenigl M, et al. COVID-19 infection in adult patients with hematological malignancies: a European Hematology Association Survey (EPICOVIDEHA). J Hematol Oncol. 2021;14(1):168.

3. Genomic Surveillance at the National Institute of Infectious Diseases [Available from: https://www.mhlw.go.jp/stf/seisakunitsuite/newpage_00061.html. Accessed 7 March 2023.

4. Changes in PANGO lineage of SARS-CoV-2 [Available from: https://www.niid.go.jp/niid/images/cepr/covid-19/20230222_genome_surveillance.pdf. Accessed 7 March 2023.

5. Puhach O, Meyer B, Eckerle I. SARS-CoV-2 viral load and shedding kinetics. Nat Rev Microbiol. 2023;21(3):147-61.

6. Rhee C, Kanjilal S, Baker M, Klompas M. Duration of Severe Acute Respiratory Syndrome Coronavirus 2 (SARS-CoV-2) Infectivity: When Is It Safe to Discontinue Isolation? Clin Infect Dis. 2021;72(8):1467-74.

7. Garcia-Vidal C, Puerta-Alcalde P, Mateu A, Cuesta-Chasco G, Meira F, Lopera C, et al. Prolonged viral replication in patients with hematologic malignancies hospitalized with COVID-19. Haematologica. 2022;107(7):1731-5.

8. Tegally H, Moir M, Everatt J, Giovanetti M, Scheepers C, Wilkinson E, et al. Emergence of SARS-CoV-2 Omicron lineages BA.4 and BA.5 in South Africa. Nat Med. 2022;28(9):1785-90.

9. Common Investigation Protocol for Investigating Suspected SARS-CoV-2 Reinfection [Available from: https://public4.pagefreezer.com/browse/CDC%20Covid%20Pages/11-05-2022T12:30/https://www.cdc.gov/coronavirus/2019-ncov/php/reinfection.html Accessed 1 July 2023.

10. Hadjadj J, Yatim N, Barnabei L, Corneau A, Boussier J, Smith N, et al. Impaired type I interferon activity and inflammatory responses in severe COVID-19 patients. Science. 2020;369(6504):718-24.

11. Takashita E, Yamayoshi S, Simon V, van Bakel H, Sordillo EM, Pekosz A, et al. Efficacy of Antibodies and Antiviral Drugs against Omicron BA.2.12.1, BA.4, and BA.5 Subvariants. N Engl J Med. 2022;387(5):468-70.

12. Riester E, Findeisen P, Hegel JK, Kabesch M, Ambrosch A, Rank CM, et al. Performance evaluation of the Roche Elecsys Anti-SARS-CoV-2 S immunoassay. J Virol Methods. 2021;297:114271.

Supplementary Table 1. Baseline characteristics of enrolled patients in the entire cohort.

	**Total (n=1197)**
**Age, year, median (IQR)**	72 (63-79)
Age ≧70, n (%)	710 (59.3)
**Female, n (%)**	548 (45.7)
**Background hematologic disease, n (%)**	
Lymphoid neoplasms	792 (66.2)
Malignant lymphoma	534 (44.6)
BCL	465 (38.9)
TCL	45 (3.7)
HL	24 (2.0)
Plasma cell dyscrasia	229 (19.1)
MM	146 (12.2)
sMM/MGUS	83 (6.9)
Acute lymphoblastic leukemia	29 (2.4)
Myeloid neoplasms	298 (24.9)
AML/MDS	134 (11.2)
MPN	106 (8.8)
CML	58 (4.8)
Benign hematologic disorders	107 (8.9)
AA	32 (2.7)
Others	75 (6.2)
**Treatment, n (%)**	
Ongoing treatment	483 (40.3)
Anti-CD20 antibody within two years	234 (19.5)
Bendamustine within two years	51 (4.2)
HSCT within two years	27 (2.2)
**Vaccination dose, n (%)**	
≧three	1085 (90.6)
Two	78 (6.5)
Missing	34 (2.9)
**Preexposure prophylaxis with tixagevimab–cilgavimab, n (%)**	80 (6.7)

Abbreviations: IQR, interquartile range; BCL, B-cell lymphoma; TCL, T-cell lymphoma; HL, Hodgkin lymphoma; MM, multiple myeloma; sMM, smoldering multiple myeloma; MGUS, monoclonal gammopathy of undetermined significance; AML, acyte myeloid leukemia; MDS, myelodysplastic syndrome; MPN, myeloproliferative neoplasms; CML, chronic myeloid leukemia; AA, aplastic anemia; HSCT, hematopoietic stem cell transplantation.

Supplementary Table 2. Comparison of clinical characteristics between the original cohort and the subgroup of patients evaluated for PVS.

	**Total patients with COVID-19 (n=160 [100%])**	**Patients with PVS evaluation (n=46 [28.7%])**	**P-value**
**Age, years, median (IQR)**	68 (57-78)	73 (66-80)	0.061
≧70, n (%)	71 (44.4)	27 (58.6)	0.134
**Female, n (%)**	62 (38.8)	13 (28.2)	0.226
**ECOGPS ≧2, n (%)**	22 (13.8)	12 (35.2)	0.069
**Background hematological disease, n (%)**			
B-cell lymphoma	64 (40.0)	21 (45.6)	0.093
Plasma cell dyscrasia	28 (17.5)	9 (19.5)	0.828
Myeloid neoplasms	35 (21.9)	4 (8.7)	0.053
Others	33 (20.6)	12 (26.1)	0.42
**Treatment of underlying disease, n (%)**			
Anti-CD20 antibody within 2 years	41 (25.6)	21 (45.6)	0.011
Bendamustine within 2 years	11 (6.9)	7 (8.7)	0.133
**Vaccination doses before infection, n (%)**			
Two	43 (26.9)	16 (34.7)	0.355
≧Three	117 (73.1)	30 (65.3)	-
**Outcome, n (%)**			
Severe or critical disease	21 (13.1)	10 (21.7)	0.163
Hospitalization	34 (21.3)	18 (39.1)	0.02
Death attributable to COVID-19	4 (2.5)	2 (4.3)	0.617

Abbreviations: PVS, prolonged viral shedding; IQR, interquartile range; ECOGPS, European Cooperative Oncology Group Performance Status.

Supplementary Table 3: Univariate analysis for predicting PVS in 46 patients with COVID-19.

	Patients with PVS (n=17)	Patients without PVS (n=29)	Univariate analysis		
			Odds ratio	95% CI	P
**Age ≧70 years, n (%)**	13 (76.5)	14 (48.3)	3.48	0.97–14.75	0.067
**ECOGPS ≧2, n (%)**	7 (41.2)	5 (17.2)	3.36	0.87–13.93	0.081
**Background hematologic disease, n (%)**					
B-cell lymphoma	12 (70.6)	13 (44.8)	2.95	0.85–11.35	0.095
Plasma cell dyscrasia	3 (17.6)	6 (20.7)	0.82	0.15–3.66	0.802
Myeloid neoplasms	0 (0)	4 (13.8)	-	-	-
Others	2 (11.8)	6 (20.7)	0.51	0.06–2.56	0.446
**Vaccination number ≧3, n (%)**	10 (58.8)	20 (68.9)	0.64	0.18–2.26	0.487
**COVID-19 treatment, n (%)**	17 (100)	17 (58.6)	-	-	-
**Severe COVID-19, n (%)**	7 (41.2)	3 (10.3)	6.06	1.39–32.77	0.021
**Treatment of hematologic disease, n (%)**					
Ongoing treatment	8 (47.0)	7 (24.1)	2.79	0.78–10.38	0.114
Anti-CD20 antibody within 2 years	12 (70.6)	9 (31.0)	5.33	1.51–21.32	0.012
Bendamustine within 2 years	6 (35.3)	1 (3.4)	15.27	2.26–306.56	0.016
**Lymphocyte and subpopulation counts, n (%)**					
Low lymphocytes (< median 1000 /μL)	9 (52.9)	12 (41.4)	1.59	0.47–5.44	0.448
Low CD19+ cells (< median 10 /μL)	13 (76.5)	10 (34.5)	6.17	1.69–26.81	0.008
Low CD3+ cells (< median 800 /μL)	11 (64.7)	12 (41.4)	2.59	0.77–9.43	0.131

Low CD4+ cells (< median 300 /µL)	13 (76.5)	8 (27.6)	8.53	2.29–38.19	0.002
Low CD8+ cells (< median 400 /µL)	9 (52.9)	16 (55.2)	0.91	0.27–3.08	0.883
**Anti-S seronegative after infection, n (%)**	6 (35.3)	1 (3.4)	15.27	2.26–306.56	0.016

Abbreviations: PVS, prolonged viral shedding; ECOGPS, European Cooperative Oncology Group Performance Status; anti-S, antibody against spike receptor binding domain; COVID-19, coronavirus disease 2019; CI, confidence interval.

Supplementary Table 4. Clinical characteristics of patients evaluated for PVS, subgroup undergoing T-cell phenotyping, and healthy controls.

	Patients with PVS evaluation (n=46)		Patients with T-cell phenotypic analysis (n=17)		HCW (n=10)
	PVS (n=17)	Non-PVS (n=29)	PVS (n=7)	Non-PVS (n=10)	
**Age, years, median (IQR)**	79 (71-80)	69 (65-81)	74 (63-80)	70 (47-75)	35 (30-50)
≧70, n (%)	13 (76.5)	14 (48.3)	5 (71.4)	5 (50.0)	0 (0)
**Female, n (%)**	5 (29.4)	8 (27.6)	1 (14.2)	1 (10.0)	6 (60.0)
**ECOGPS ≧2, n (%)**	7 (41.2)	5 (17.2)	2 (28.5)	1 (10.0)	0 (0)
**Background hematological disease, n (%)**					
B-cell lymphoma	12 (70.6)	13 (44.8)	6 (85.7)	4 (40.0)	-
Plasma cell dyscrasia	3 (17.6)	6 (20.7)	1 (14.2)	3 (30.0)	-
Myeloid neoplasms	0 (0)	4 (13.8)	0 (0)	0 (0)	-
Others	2 (11.8)	6 (20.7)	0 (0)	3 (30.0)	
**Treatment of underlying disease, n (%)**					
Anti-CD20 antibody within 2 years	12 (70.5)	9 (31.0)	5 (71.4)	2 (20.0)	-
Bendamustine within 2 years	6 (35.3)	1 (3.4)	3 (42.8)	1 (10.0)	-
**Vaccination doses before infection, n (%)**					
Two	7 (41.2)	9 (31.0)	2 (28.5)	1 (10.0)	0 (0)
≧Three	10 (58.8)	20 (69.0)	5 (71.4)	9 (90.0)	10 (100)
**Lymphocyte and its subset counts, /µL (IQR)**					
Lymphocyte count	972 (602-1364)	1078 (900-1755)	874 (602-1168)	788 (600-2194)	-
CD19+ cell count	0 (0-9)	37 (0-157)	0 (0-4)	44 (1-279)	-
CD3+ cell count	655 (247-942)	895 (668-1290)	499 (274-580)	707 (420-1405)	-
CD4+ cell count	174 (75-290)	409 (216-555)	159 (80-174)	319 (154-839)	-

CD8+ cell count	399 (161-622)	368 (258-673)	349 (172-427)	297 (224-466)	-
**Outcome, n (%)**					
Severe or critical disease	7 (41.2)	3 (10.3)	4 (57.1)	1 (10.0)	0 (0)
Hospitalization	12 (70.6)	6 (20.7)	6 (85.7)	2 (20.0)	0 (0)
Death attributable to COVID-19	2 (11.7)	0 (0)	1 (14.2)	0 (0)	0 (0)

Abbreviations: PVS, prolonged viral shedding; HCW, health care worker; IQR, interquartile range; ECOGPS, European Cooperative Oncology Group Performance Status.

Supplementary Table 5. Detailed chemotherapy and immunological status in patients with a previous history of anti-CD20 antibody and bendamustine treatment.

Pt.	Age	HD	Cumulative dose of Benda (mg/m^2)	Time from Benda (month)	Time from mCD20ab (month)	Vaccine dose	Anti-S (U/mL)	CD4+ (/μL)	PVS
1	80	FL	900	1	1	2	3.6	16	Yes
2	68	FL	720	19	4	4	0.4	42	NA
3	74	FL	720	1	1	2	0.4	174	Yes
4	73	WM	450	32	3	2	0.4	441	NA
5	49	DLBCL	430	1	1	2	211	13	Yes
6	81	MCL	860	40	7	4	0.4	297	NA
7	81	FL	720	62	18	4	0.4	159	Yes
8	68	FL	720	95	15	4	62.3	585	Yes
9	79	FL	500	13	13	3	0.4	357	Yes
10	80	FL	900	5	1	4	0.4	174	Yes
11	82	LGBL	280	0	0	3	1007	55	Yes
12	71	WM	600	128	0	3	618	411	NA
13	74	FL	970	27	5	3	0.4	168	NA
14	80	FL	900	51	3	2	1.1	384	Yes
15	66	FL	1080	27	5	4	0.4	79	No

16	66	MALT	1080	13	13	3	3.2	142	No
17	73	MALT	720	13	13	4	0.4	36	NA

Abbreviations: Pt., patient; HD, hematologic disease; Benda, bendamustine; FL, follicular lymphoma; WM, Waldenström macroglobulinemia; DLBCL, diffuse large B-cell lymphoma; MCL, mantle cell lymphoma; LGBL, low-grade B-cell lymphoma; MALT, mucosa-associated lymphoid tissue lymphoma; mCD20ab, monoclonal anti-CD20 antibodies; Anti-S, anti-SARS-CoV-2 spike receptor binding domain antibodies; PVS, prolonged viral shedding; NA, not assessed.

Supplementary Figure 1: Flow diagram of enrollment for assessment of viral dynamics, T-cell phenotypic analysis, and neutralizing activities against virus subvariants.

Abbreviations: RT-PCR, reverse transcription-polymerase chain reaction; Ct, cycle threshold; PVS, prolonged viral shedding; HCW, health care worker.

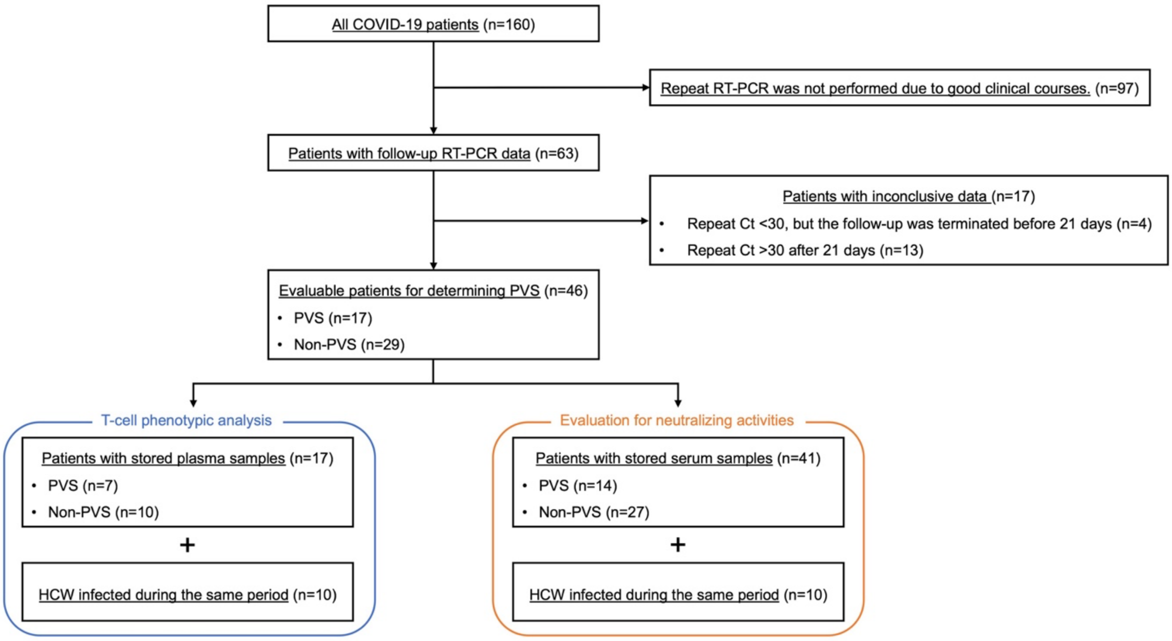

Supplementary Figure 2: Swimmer plot of the clinical course of COVID-19 in patients previously treated with bendamustine and anti-CD20 antibodies. Time course and specific treatments, accompanying symptoms, and outcomes are summarized.

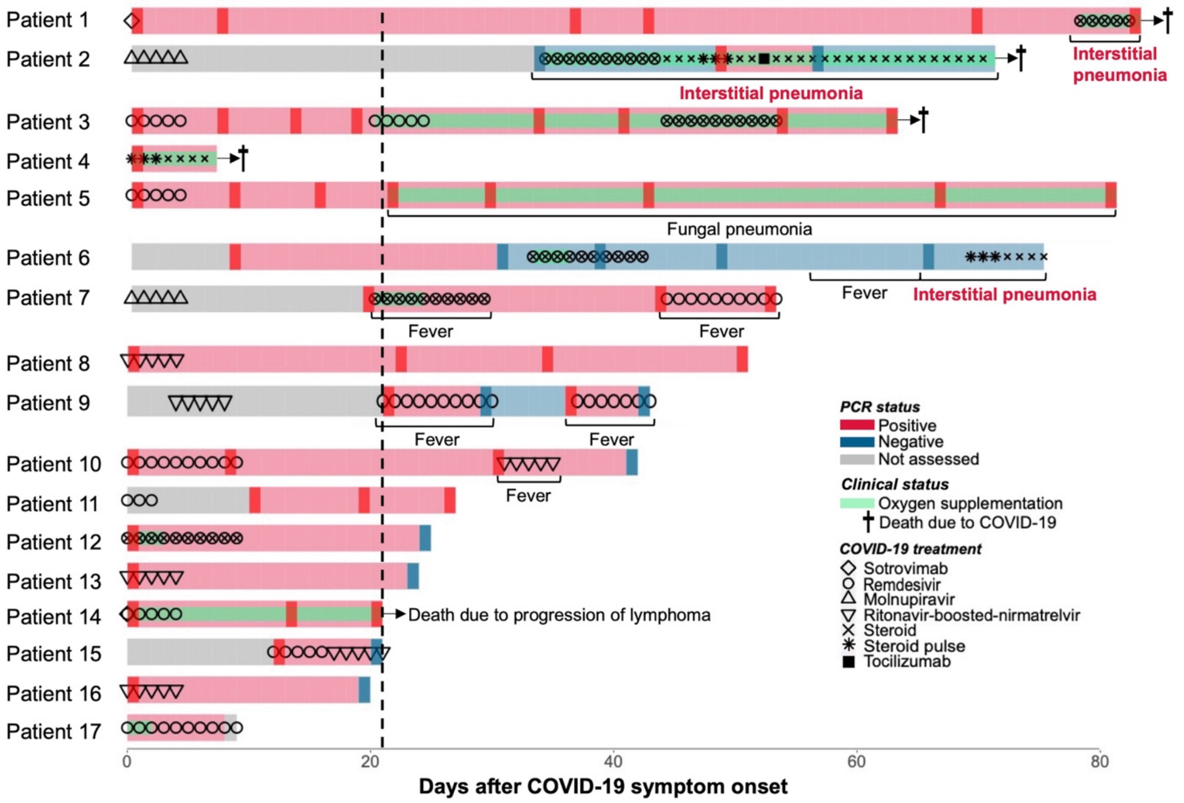

Supplementary Figure 3: Radiological and pathological findings of patients who developed fatal late-onset interstitial pneumonia. The upper panels show the radiological changes in chest computed tomography between onset and before death. Localized ground-grass opacities progressed bilaterally to an extended area accompanied by strong traction bronchiectasis. The lower panels show the autopsy findings of the lungs, with extensive organizing pneumonia and pulmonary fibrosis combined with severe diffuse alveolar damage. Abbreviations: H&E, hematoxylin and eosin staining; M&T, Masson's trichrome staining.

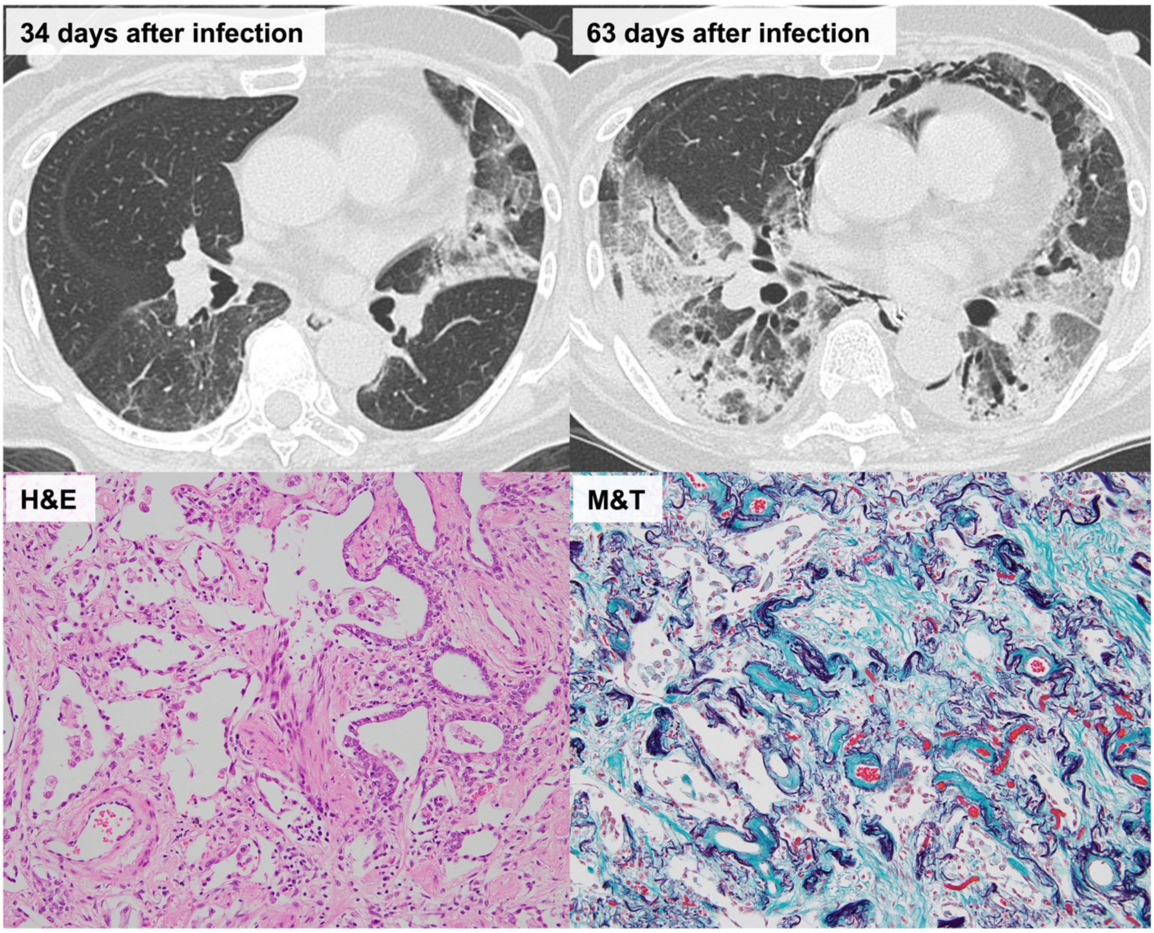

Supplement: Supplementary file 1 — Supplemental materials [file 41408_2023_897_MOESM1_ESM.pdf]
